# The Interpretation of the Term "Extractum Belladonnæ."

**Published:** 1904-06-25

**Authors:** 


### The Interpretation of the Term "Extractum Belladonnæ."

It appears that in certain pharmaceutical circles some difference of opinion and practice has recently arisen in reference to the interpretation of the term "Ext. Belladonnæ" when this appears in physicians' prescriptions. The responsibility for this uncertainty must be placed, at least in part, on the shoulders of the compilers of the last edition of the British Pharmacopœia. Until the date of the issue of this edition the Pharmacopœia of 1885 had official force, and in this, "Ext. Belladonnæ" was an extract obtained from the fresh leaves of the belladonna plant and commonly known as the "green" extract. When the 1898 volume came into existence this same preparation appeared under the style and title of "Ext. Belladonnæ Viride" and the name "Ext. Belladonnæ" had disappeared from the official record. Further, two other extracts made from the belladonna plant were named and recognised. The one "Ext. Belladonnæ Liquidum," the other "Ext. Belladonnæ Alcoholicum," each of these being obtained from belladonna root by the agency of an alcoholic menstruum. The position, therefore, under the present Pharmacopœia is, that the name "Ext. Belladonnæ" has no official recognition or interpretation. That is, the authority which professes to be and ought to be "one uniform standard and guide whereby the nature and composition of substances to be used in medicine may be ascertained and determined" is neither a standard nor a guide so far as the phrase "Ext. Belladonnæ" is concerned. The pharmacist who finds this phrase in a prescription turns in vain to the pages of the Pharmacopœia for a precise and confident interpretation. Considering how frequently the term is used in prescriptions and how for many years it was applied with pharmacopœial sanction to a definite preparation, namely the "green" extract, this seems a very absurd position. And it could have been entirely avoided by placing the term "Ext. Belladonnæ" as a synonym for the preparation now known officially as "Ext. Belladonnæ Viride." The two names in successive Pharmacopœias refer essentially to one and the same thing, and it would have avoided all possibility of ambiguity and confusion had the older term been carried forward as a synonym into the new volume. Unfortunately there are certain authorities concerned in the construction of the Pharmacopœia who forget that this exists not for the display of a pedantic passion for a severely accurate and symmetrical nomenclature, but for the public interest and safety. It is this influence, we doubt not, that is responsible for the fact that in the volume sanctioned by Parliament for the authoritative interpretation of the terms used in physicians' prescriptions the phrase "Ext. Belladonnæ" has neither part nor lot. Nevertheless, the duty of the pharmacist seems to us free from doubt. "Ext. Belladonnæ," it is true, is a term for which the existing Pharmacopœia offers no explanation, and it is therefore open to the pharmacist to request the prescriber to define the preparation he desires. But in view of all the circumstances this may be held to be quite unnecessary. Until 1898 the term had an official existence and was the official name of the "green" extract. The "green" extract still exists, though it is now officially qualified as "viride." It cannot be doubted that prescribers in the present year of grace mean by "Ext. Belladonnæ" the preparation so named until the issue of the Pharmacopœia of 1898. This is the "green" extract. And pharmacists may safely adhere to the principle that when the physician uses a term which is not in the existing Pharmacopœia but was recognised and defined in the immediately preceding Pharmacopœia, he means the preparation thus recognised and defined. Every one of the official (1898) extracts of belladonna has now a specific qualification—"alcoholic," "liquid," or "green"—and therefore when "extract" is written without qualification there is on the present-day facts room for uncertainty. But when all the circumstances are duly considered the pharmacist may, we feel sure, rely on the interpretation we have ventured to suggest. If his conscience is perturbed he has the option of consulting the prescriber. And for any uncertainty which prescriber or dispenser may suffer, thanks are to be rendered to the strict disciplinarians who have played the schoolmaster in the pages of the British Pharmacopœia.

